# Extreme fetal macrosomia at 42 gestational weeks: a case report and literature review

**DOI:** 10.1515/crpm-2021-0042

**Published:** 2022-06-27

**Authors:** Julia Kummer, Yvonne Callister, Anja Jebens, Valentin Mihajlov, Luisa Maria Pech, Lars Hellmeyer

**Affiliations:** Department of Gynecology and Obstetrics, Klinikum im Friedrichshain, Berlin, Germany

**Keywords:** fetal macrosomia, gestational diabetes mellitus, maternal obesity, unsupervised pregnancy

## Abstract

**Objectives:**

Fetal macrosomia is a term to describe excessive fetal birth weight. Fetal macrosomia is strongly associated with adverse obstetrical outcome.

**Case presentation:**

We report a rare case of excessive neonatal weight in a medically unsupervised pregnancy and give a literature review on this significant subject. A 38 year-old woman (Gravida 8 Para 7) presented herself at 42 2/7 weeks of gestation at the labor ward. The pregnancy had not been supervised medically. Labor induction was initiated. Due to failure to progress and suspicion of a disproportion of the fetal head and maternal pelvis an urgent caesarean section was performed. A morbidly macrosomic male infant was delivered (birth weight: 6,760 g [>99. percentile], length: 60 cm [>99. percentile]).

**Conclusions:**

The morbidity for infants and women increases with a birth weight exceeding 4,500 g. Gestational diabetes mellitus, a high pre-pregnancy body mass index and excessive gestational weight gain have been independently associated as risk factors. The increase in pregnancies complicated by maternal obesity and gestational diabetes emphasizes the necessity of evidence-based clinical interventions to prevent or reduce these diseases. If prenatal care is not frequented by mothers-to-be there are no options open for obstetricians to detect fetal macrosomia and to intervene.

## Introduction

Fetal macrosomia is a term to describe excessive fetal birth weight [1]. Fetal macrosomia is strongly associated with adverse obstetrical outcome [1, 2]. The morbidity for infants and women increases with a birth weight exceeding 4,500 g [3]. Gestational diabetes mellitus, a high pre-pregnancy body mass index and excessive gestational weight gain have been independently associated as risk factors [2]. According to the ISUOG Guidelines indispensable elements of antenatal care include the screening for and management of fetal growth abnormalities [4]. Predicting macrosomia correctly and establishing a therapeutic regimen help optimizing both perinatal and maternal outcome [5]. So far it is still object to ongoing research to find an accurate way of predicting fetal macrosomia and establish interventions to prevent it [5]. We report a unique case of excessive neonatal weight in a medically unsupervised pregnancy.

## Case presentation

We report a case of excessive neonatal weight in a medically unsupervised pregnancy and give a literature review on the subject.

A 38 year-old woman (Gravida 8 Para 7) presented herself for the first time at 42 2/7 weeks of gestation at the labor ward for a check-up. The patient had a history of seven spontaneous vaginal deliveries with neonates weighing between 3,100 g and 4,800 g.

This pregnancy had not been supervised medically except for one check-up in 32 weeks of gestation. Blood pressure was taken and showed 148/85 mmHg, heart rate 111 bpm, temperature 36.5 °C. The woman was obese with a body mass index of 46.8 kg/m^2^. Since the patient had not seeked medical consultation before, pre-existing maternal diseases had not been diagnosed so far.

Fetal heart rate patterns were monitored and showed a normal pattern with a normal oscillation, sporadic accelerations and no decelerations. The ultrasound examination revealed a vital macrosomic singleton pregnancy with an estimated weight of 4,100 g (estimation formula generated by Hadlock). However, assessibility was extremely limited due to maternal obesity.

Labor induction was induced with oxytocin under close supervision of the maternal blood pressure. A spontaneous rupture of the membranes occurred with clear amniotic fluid leaking in the early stage of labor. Due to her obesity epidural anesthesia could not be performed so the patient received analgesia with an opioid. During the second stage of labor it came to failure to progress due to fetal malposition with persistent occiput posterior position and suspicion of a disproportion of the fetal head and maternal pelvis. An urgent caesarean section was performed in general anesthesia and a vital morbidly macrosomic male infant was delivered (birth weight: 6,760 g [>99. percentile], length: 60 cm [>99. percentile], APGAR 4-8-9, NA-pH: 7.15, BE -5.3 mmol/L).

The patient received cefuroxime as a single shot and carbetocin for uterus contraction initially. Due to an atonic uterus medication was switched to sulprostone. Blood loss in total during the caesarean section was 1,500 mL. The patient’s vital parameters were stable at all times.

Due to tachydyspnea the baby boy required face mask-delivered non-invasive ventilation from birth until 24 h of life. The neonate was transferred to the neonatal intensive care unit where a chest X-ray was performed. It showed only minor regional ventilation dysfunction. Blood was drawn and yielded a normal white cell count and an elevated C-reactive protein level (results presented in Table 1) so that intravenous antibiotic treatment with ampicillin and gentamicin was initiated. Since blood cultures were negative for bacteria and infectious parameters were dropping the antibiotic treatment was terminated after five days.

**Table 1:** Neonatal laboratory values.

Parameter	Value	Reference range	Unit
Leukocytes	10.45	7.20–21.60	/nl
Hemoglobin	19.1	13.2–21.7	g/dl
Platelets	183	220–520	/nl
Sodium	140	131–144	mmol/L
Potassium	5.5	3.2–5.5	mmol/L
Interleukin-6	172.7	<30.4	ng/l
C-reactive protein	30.4	<5.0	mg/L
Glucose	33		mg/dl
Thyroid stimulating hormone	1.97	2.12–13.50	mU/l
Free thyroxine	25.90	10.60–23.00	ng/l
Total triiodothyronine	3.90	2.16–5.88	ng/l
Insulin-like growth factor 1	50.9	19.0–130.0	ng/ml
Adrenocorticotropic hormone	35.7	10.0–185.0	pg/ml
Cortisol	71.6	80.0–300.0	nmol/l

The neonate initially developed hypoglycemia with a blood sugar level of 33 g/dL. He needed oral glucose supplementation once and in addition to breast-feeding supplementary formula-feeding.

An extensive diagnostic regimen was initiated due to neonatal macrosomia. Clinically the baby boy presented no signs of a syndromal disorder. Blood was drawn to examine the function of the neonate’s hypothalamic-pituitary-thyroid axis. The results are demonstrated in Table 1. Except for elevated thyroxine (fT4) and low thyroid-stimulating hormone (TSH) there were no pathological findings.

**Figure 1:**
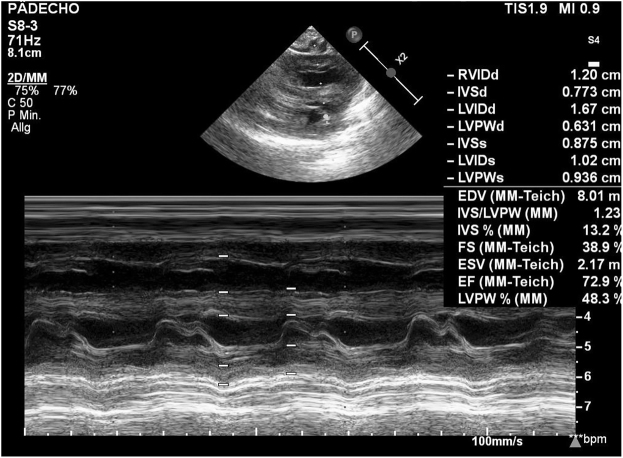
Neonatal echocardiography.

Echocardiography was performed and showed a generalized myocardial hypertrophy, a patent ductus arteriosus and a small patent foramen ovale (Figure 1).

**Figure 2:**
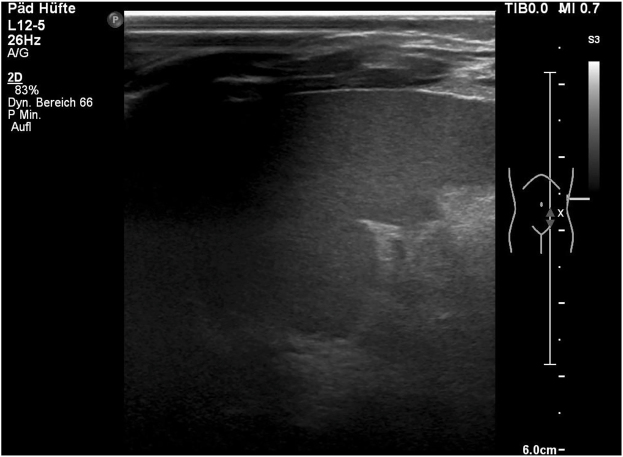
Neonatal abdominal ultrasound showing excessive abdominal fat.

Apart from excessive abdominal fat abdominal ultrasound yielded no signs of congenital malformations or tumorous alterations. Cerebral ultrasound showed normal results (Figure 2).

At maternal urging the neonate was discharged on the 11th day of living against medical advise. The neonate was scheduled for a postdischarge follow-up to which mother and child did not appear.

The maternal postoperative course included a detailed blood check-up which showed an anemia and elevated HbA1c of 7.2% (results presented in Table 2). Blood sugar levels were monitored closely and demonstrated elevated values. However, blood sugar levels did not make insulin treatment necessary. Oral iron supplementation was initiated.

**Table 2:** Postoperative maternal laboratory values.

Parameter	Value	Reference range	Unit
Leukocytes	15.15	3.90–10.50	/nl
Hemoglobin	7.3	12.0–15.6	g/dl
Platelets	268	150–370	/nl
Sodium	139	136–145	mmol/L
Potassium	4.1	3.4–4.5	mmol/L
Creatinine	0.79	0.50–0.90	mg/dl
Glutamate oxaloacetate transaminase	29	<35	U/l
Glutamate pyruvate transaminase	16	<31	U/l
C-reactive protein	21.7	<5.0	mg/L
Lactate dehydrogenase	422	135–250	U/l
Glycated hemoglobin	7.2	<6.0	%
Glucose	167	74–106	mg/dl
Thyroid stimulating hormone	3.22	0.27–4.20	mU/l
Prolactin	309.00	4.79–23.30	µG/l
Human growth hormone	0.09	<5.00	mg/ml
Insulin-like growth factor 1	175.2	72.0–237.0	mg/ml
Cortisol	579.0		mmol/L
Estradiol	206.0	46.0–1828.0	pmol/l

The maternal blood pressure was monitored closely and showed elevated results. An antihypertensive medication with alpha-methyldopa was initiated which normalized maternal blood pressure.

The patient underwent a neurosurgical examination which ruled out acromegaly clinically. Additionally laboratory results showed no overproduction of insulin-like growth factor 1 (IGF-1) (results shown in Table 2).

In summary of all findings the neonate’s excessive weight was led back to either a pre-existing or gestational maternal unsupervised diabetes mellitus and extreme maternal adiposity.

## Discussion

Excessive neonatal birth weight with its increasing incidence complicates obstetrical management and outcome severely. The proportion of fetal macrosomia ranges from 5 % to 20% in high income countries according to studies worldwide with a tendency of increase over the last decades [2].

Predicting fetal macrosomia correctly and establishing a therapeutic regimen could help to optimize both perinatal and maternal outcome [5]. Strategies of predicting fetal macrosomia consist of three major aspects: assessing clinical risk factors, performing Leopold’s maneuvers and using ultrasound [5].

According to Gaudet et al. risk factors for excessive fetal birth weight include maternal pre-existing and gestational diabetes mellitus, previous macrosomic birth, postterm gestation past 42 weeks of gestation and male infant gender [1]. Of the aformentioned maternal diabetes mellitus is the strongest risk factor [5]. Gestational diabetes defined as a glucose intolerance with an onset or first recognition during pregnancy increases the risk for fetal macrosomia to a 2-fold [2, 5]. International guidelines recommend the use of the 75 g 2 h oral glucose tolerance test to test for gestational diabetes in women with risk factors and to establish early interventions to prevent gestational diabetes mellitus related complications [6].

The meta-analysis of Gaudet et al. additionally states a strong association between fetal macrosomia and maternal obesity [1]. The underlying mechanisms are not yet understood completely but seem to be associated with a dysregulation of glucosis, insulin, lipid and amino acid metabolism, both in mother and child [2]. This implicates the necessity of preventing maternal overnutrition and monitoring maternal weight during pregnancy strictly [7]. Pre-pregnancy obesity is associated with a 1.6-fold increase in risk for neonatal macrosomia [7]. Optimization of the maternal weight prior to the pregnancy through the encouragement of lifestyle modifications including diet control and exercise seem essential in managing gestational weight gain and abnormal glucosis metabolism [1, 7]. Excessive weight gain during pregnancy increases the risk for neonatal macrosomia 3.6 times compared to women with normal weight gain during pregnancy [7].

Genetic conditions need to be considered as differential diagnosis for fetal overgrowth as well [8]. Overgrowth syndromes like Beckwith-Wiedemann, Pallister-Killian, Sotos or Perlman are often associated with tumors, developmental delays or other abnormalities and can therefore be relevant for pediatric neonatal care [8]. Although the occurrence of overgrowth syndromes is rare prenatal ultrasound in macrosomic fetuses should check for brain abnormalities, heart defects, sceletal anomalies, abdominal wall defects and visceral organ anomalies [8].

In our case two major risk factors contributed to fetal macrosomia. On the one hand the patient was extremely obese with a body mass index of 46.8 kg/m^2^. On the other hand advanced maternal insulin resistence had to be acknowledged. A pre-existing diabetes mellitus can be suspected.

Most common biometric ultrasound measurements to estimate fetal weight involve biparietal diameter, head circumference, abdominal circumference and femur diaphysis length [4, 9].

Nevertheless, the accurate detection of fetal macrosomia via ultrasound is limited [4, 9]. Clinical decisions concerning fetal macrosomia based on ultrasound prediction only need to be questioned [9].

Maternal complications caused by fetal macrosomia include emergency caesarean section, peripartum hemorrhage and anal sphincter injury [3]. In our case the patient received an urgent caesarean section due to failure to progress in the second stage of labor with a persistent occiput posterior position of the fetus. Even though the patient quickly received uterotonic agents we experienced a postpartum hemorrhage with a total blood loss of 1,500 mL and a consecutive maternal anemia. The systemic review of eight studies by Beta et al. showed a 2,5-fold increase in the performance of emergency caesarean sections in pregnancies with a neonatal birth weight of >4,500 g compared to those without macrosomic neonates [3].

Beta et al. were able to show that pregnancies with a neonatal birth weight of >4,000 g had a 2-fold increased risk of peripartal hemorrhage and pregnancies with a neonatal birth weight of >4,500 g a 3-fold increased risk compared to pregnancies with non-macrosomic neonates [3].

Compared to non-macrosomic pregnancies there is a significant increase in obstetric anal sphincter injury in pregnancies with a neonatal weight >4,000 g, especially when complicated by shoulder dystocia [3].

So far, there exist no established guidelines on how to inform and advise women when fetal macrosomia is suspected [3].

Neonatal outcome is severely impaired due to macrosomic birth weight as well [3]. Macrosomic newborns form a heterogenous patient group in regard to body constitution and metabolism [2]. An increased birth weight is however associated with a 2–3-fold increase in risk of intrauterine death [2]. Macrosomic newborns present an increased risk of prolonged neonatal intensive care, especially in the presence of maternal diabetes mellitus as well as when weighing more than 5,000 g, having shown fetal distress or when suspecting a cephalopelvic disproportion [10]. All of the aformentioned applied in our case.

Macrosomic birth weight furthermore augments the risk of shoulder dystocia, obstetric brachial plexus injury and clavicular fracture in newborns after vaginal delivery significantly [3].

Lately, more attention has been drawn to the long-term consequences on childrens’ health due to maternal obesity [11]. Animal models were able to show unambiguously that maternal obesity promotes insulin resistance in the offspring as well as the development of cardiovascular disease risk factors later on in life [11]. However, human observational studies have not yet distinguished causality from association concerning maternal obesity and childhood diseases due to the complexity of influential factors and confounder data [11].

The increase in pregnancies complicated by maternal obesity and gestational diabetes emphasizes the necessity of evidence-based clinical interventions to prevent or decelerate these diseases. However, if prenatal care is not frequented by mothers-to-be there are no options open for obstetricians to intervene. A variety of studies show that fetal macrosomia complicates pregnancies and labor which we demonstrated with this case report as well.
